# Data set of optimal parameters for colorimetric red assay of epoxide hydrolase activity

**DOI:** 10.1016/j.dib.2016.05.075

**Published:** 2016-06-03

**Authors:** Gabriel Stephani de Oliveira, Patricia Pereira Adriani, Flavia Garcia Borges, Adriana Rios Lopes, Patricia T. Campana, Felipe S. Chambergo

**Affiliations:** a Escola de Artes, Ciências e Humanidades, Universidade de São Paulo, Av. Arlindo Béttio, 1000, São Paulo, Brazil; b Instituto Butantan, Av. Vital Brasil, 1500, São Paulo, Brazil

**Keywords:** Colorimetric red assay, Epoxide hydrolase, Kinetic parameters

## Abstract

The data presented in this article are related to the research article entitled “Epoxide hydrolase of *Trichoderma reesei*: Biochemical properties and conformational characterization” [1]. Epoxide hydrolases (EHs) are enzymes that catalyze the hydrolysis of epoxides to the corresponding vicinal diols. This article describes the optimal parameters for the colorimetric red assay to determine the enzymatic activity, with an emphasis on the characterization of the kinetic parameters, pH optimum and thermal stability of this enzyme. The effects of reagents that are not resistant to oxidation by sodium periodate on the reactions can generate false positives and interfere with the final results of the red assay.

## **Specifications Table**

**Table:** 

Subject area	Biology, Chemistry
More specific subject area	Protein purification and Enzimatic colorimetric assay
Type of data	Image, graph
How data was acquired	Electrophoresis and colorimetric red assay
Data format	Purified, analyzed
Experimental factors	Protein purification and dialysis of epoxide hydrolase from *Trichoderma reesei*
Experimental features	Enzimatic colorimetric assay, kinetic parameters, pH optimum and thermal stability
Data source location	University of São Paulo, São Paulo, Brazil
Data accessibility	Data is presented in this article

## **Value of the data**

• The data set described here provide optimized parameters for application of the colorimetric red assay to determine the kinetic parameters, pH optimum and thermal stability of epoxide hydrolases.
• The data set provide guidelines for the use of other epoxide hydrolases in the colorimetric red assay.

## 1 Data

**Fig. 1:**
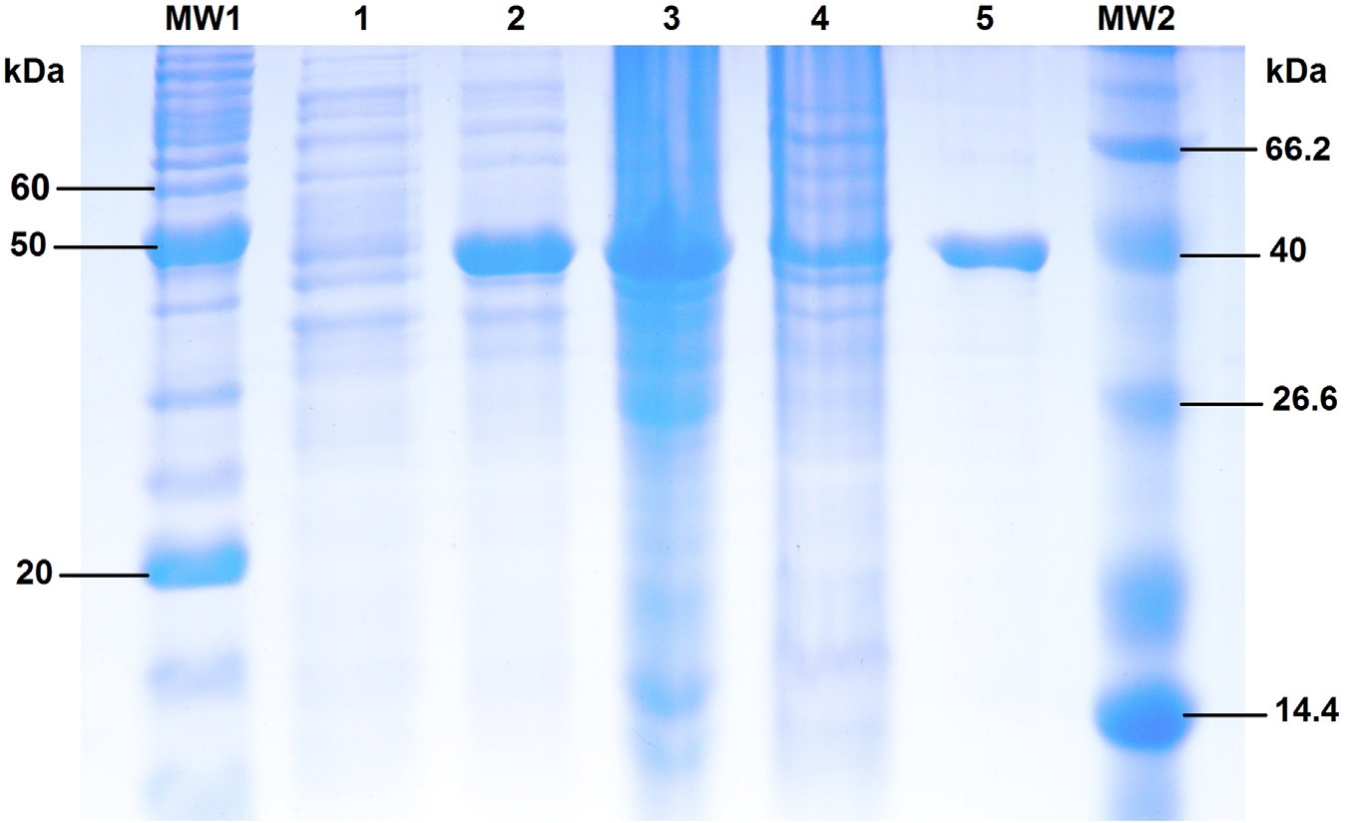
SDS-PAGE gel (12.5%) of the expression and purification of the recombinant TrEH protein. (1) Bacterial culture not induced with IPTG; (2) bacterial culture induced with IPTG; (3) bacterial lysate (not soluble); (4) bacterial lysate (soluble); (5) protein collected after purification; (MW1) molecular weight BenchMark Protein Ladder (Invitrogen, USA); (MW2) molecular weight Mid-Low Range (J450) (Amresco, USA).

**Fig. 2:**
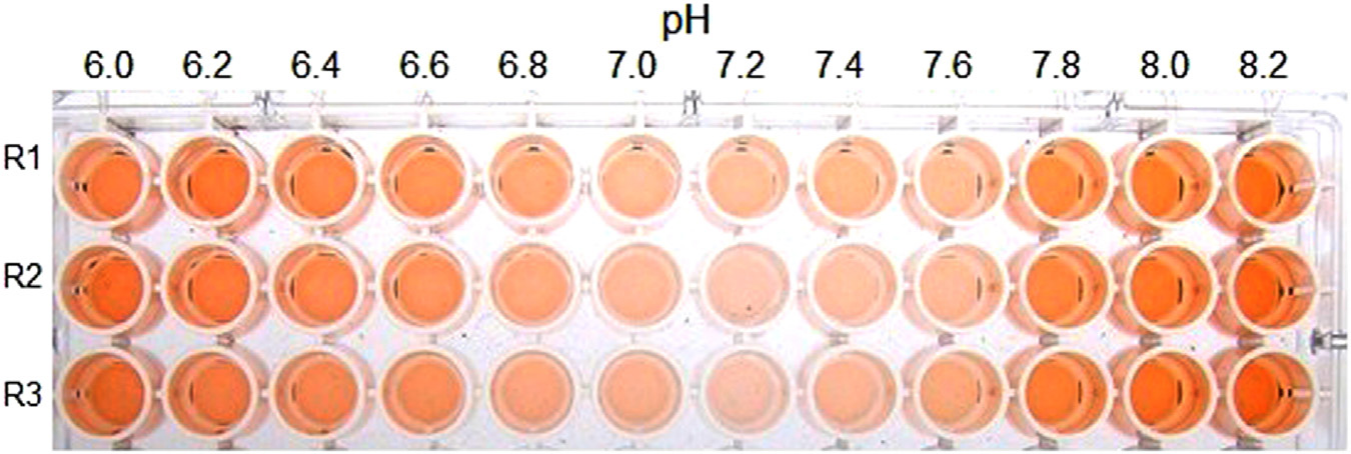
Assay used to determine the pH optimum of TrEH. Red assay conducted with TrEH (40 μg/mL) between pH 6.0 and 8.2. Rows R1, R2, and R3 show triplicate enzymatic reactions at various pHs (the pHs of the enzymatic reactions are shown above each column). The analysis of this figure shows that the highest activity of ThEH occurred between pH 7 and 7.4 because these wells display the brightest color.

**Fig. 3:**
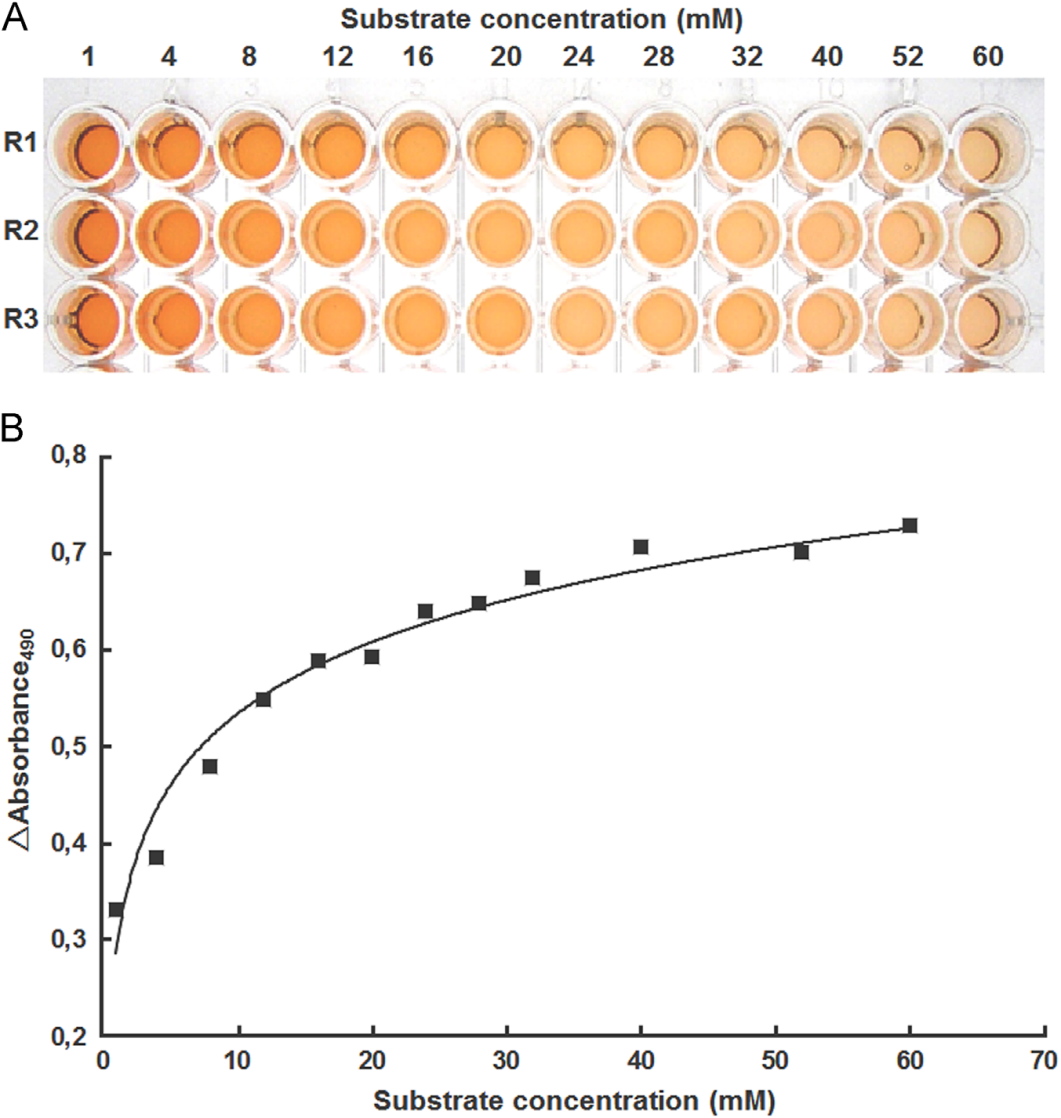
Assay used to determine the kinetic parameters of the TrEH enzyme. (A) Red assay conducted with TrEH (40 μg/mL) in sodium phosphate buffer. Rows R1, R2, and R3 show triplicate enzymatic reactions with different concentrations of the racemic styrene oxide substrate (the substrate concentrations used in the enzymatic reactions are shown above each column). (B) Graph plotting the data of △Absorbance_490_ of the red assays against the different substrate concentrations. The analysis of these figures has established that the enzyme exhibits a behavior corresponding to that described by Michaelis–Menten equation.

Here, we describe the optimal parameters for the colorimetric red assay used in determination of activity of epoxide hydrolase enzyme. See Fig. 1, Fig. 2, Fig. 3.

## 2 Experimental design, materials and methods

The purification of *Trichoderma reesei* epoxide hydrolase (TrEH) protein described by [1] was confirmed by SDS-PAGE (12.5%) (Fig. 1). The collected purified fraction was dialyzed five times against appropriate buffer solution (see above) in cellulose dialysis tubing with a molecular weight cut off of 12 kDa before the subsequent enzymatic characterization tests were performed.

The red assay used to determine the enzymatic activity of epoxide hydrolase is based on the oxidation of adrenaline by sodium periodate (NaIO_4_), which results in the formation of adrenochrome to produce the red reaction [2]. However, in the presence of the epoxide hydrolase enzyme, diol product will be formed, which can be oxidized by sodium periodate to prevent the formation of adrenochrome. Therefore, the resulting color reaction will become clearer in the same proportion as the diols are formed by the reaction with EH enzyme. The absorbance can be read at 490 nm and is an indirect measure of enzyme activity.

This assay was performed to characterize the kinetic parameters, pH optimum and thermal stability of the TrEH enzyme. Ninety microliters of the purified enzyme solution were added to each well, along with an appropriate buffer for the experiment and 10 µL of the substrate solution (styrene oxide (racemic or enantiopure) diluted in acetonitrile at a concentration suitable for each assay). The samples were incubated at 37 °C for 10 min, with the exception of the thermal stability tests. The enzymatic reaction was stopped by adding 50 µL of sodium periodate solution (5 mM NaIO_4_ diluted in 90% acetonitrile). After one hour at room temperature, the remaining sodium periodate was titrated by the addition of 50 µL of adrenaline solution (6 mM epinephrine diluted in 10% acetonitrile). The absorbance was read at 490 nm after 15 min.

The enzyme activity was calculated by the changes in absorbance (△Absorbance_490_) of the enzymatic reaction and its controls. The △Absorbance_490_ is the difference between the value of oxidant consumed by enzyme solution and diol [Absorbance of the substrate control (A_490_SC) (substrate+buffer)−Absorbance o the enzymatic reaction (A_490_R) (substrate+enzyme+buffer)] and the value of oxidant consumed by the enzyme solution [Absorbance of the buffer control (A_490_BC) (just buffer)−Absorbance of the enzyme control (A_490_EC) (enzyme+buffer)]. The adrenaline and sodium periodate solutions were added to all controls and to the reaction. The enzyme activity was calculated using the slope of the calibration curve (varying concentrations of the diol).

Because the red assay does not result in false positives, all of the components in the reaction must be resistant to oxidation by the sodium periodate solution, thus ensuring that only the diol (product of enzymatic reaction) is oxidized. To optimize the method, red assays were performed with lysed bacterial cells (without protein purification), TrEH purified fractions without dialysis and dialyzed against Tris–HCl buffer (50 mM, pH 7.5), sodium phosphate buffer (50 mM Na_2_HPO_4_/NaH_2_PO_4_, pH 7,5) and PBA buffer (20 mM sodium acetate/20 mM sodium borate/20 mM sodium phosphate monobasic). The reactions containing the purified and dialyzed TrEH against sodium phosphate buffer or PBA buffer showed that the assay was optimized and that there were no false positives. Based on these conditions, the details of the procedures used to characterize the kinetic parameters, pH optimum and thermal stability of the TrEH enzyme and others EHs are described below.

The test for determining the pH optimum of TrEH enzyme was performed with the purified enzyme (40 μg/mL) in PBA buffer between pH 6.0 and 8.2, with racemic styrene oxide (400 mM) as the substrate. This experiment showed that the enzyme has higher activity between pH 7 and 7.4 because these wells display the brightest color (Fig. 2). The red assay can be performed between pH 2 and 10, but when it is performed with the substrate styrene oxide, it cannot be performed in the more acidic pH ranges than pH 5 because styrene oxide is not stable under these conditions.

The assays used to determine the kinetic parameters of enzyme under study were also conducted using the red assay and samples of purified TrEH (40 μg/mL) in sodium phosphate buffer, which allowed us to characterize the kinetics of TrEH enzyme with racemic styrene oxide (400 mM) as the substrate, and its Michaelian behavior (Fig. 3).

To determine the thermal stability, samples of purified TrEH (40 μg/mL) in sodium phosphate buffer were incubated at 23 °C, 37 °C, 50 °C, 60 °C, and 70 °C for 20 min in thermostated circulating water bath. Aliquots were removed at 5 min intervals (0, 5, 10, 15, and 20 min) to assay the remaining activity using racemic styrene oxide (400 mM) as the substrate.
