# Supplementary material for: Data set of optimal parameters for colorimetric red assay of epoxide hydrolase activity
Source: Data Brief. 2016 Jun 3;8:436–40. doi: 10.1016/j.dib.2016.05.075 (PMC4910289; doi:10.1016/j.dib.2016.05.075)
Supplement: Supplementary file 1 — Supplementary material [file mmc1.doc]

Ms. Ref. No.: DIB-D-16-00379

Title: Data set of optimal parameters for colorimetric red assay of epoxide hydrolase activity

**Conflict of Interest Form - Data in Brief**

The authors declare that there are no conflicts of interest.

Sincerely Yours,

Felipe Chambergo A. Dr.
